# DNA methylation and cancer incidence: lymphatic–hematopoietic versus solid cancers in the Strong Heart Study

**DOI:** 10.1186/s13148-021-01030-8

**Published:** 2021-02-25

**Authors:** Arce Domingo-Relloso, Tianxiao Huan, Karin Haack, Angela L. Riffo-Campos, Daniel Levy, M. Daniele Fallin, Mary Beth Terry, Ying Zhang, Dorothy A. Rhoades, Miguel Herreros-Martinez, Esther Garcia-Esquinas, Shelley A. Cole, Maria Tellez-Plaza, Ana Navas-Acien

**Affiliations:** 1 Department of Environmental Health Sciences, Columbia University Mailman School of Public Health, New York, NY USA; 2 Department of Chronic Diseases Epidemiology, National Center for Epidemiology, Carlos III Health Institute, Melchor Fernandez Almagro Street, 5, Madrid, Spain; 3 Department of Statistics and Operations Research, University of Valencia, Valencia, Spain; 4 The Population Sciences Branch, National Heart, Lung, and Blood Institute, National Institutes of Health, Bethesda, MD USA; 5 The Framingham Heart Study, Framingham, MA USA; 6 Population Health Program, Texas Biomedical Research Institute, San Antonio, TX USA; 7 Department of Pathology, Universidad de La Frontera, Temuco, Chile; 8 Department of Mental Health, Johns Hopkins University, Baltimore, MD USA; 9 Department of Epidemiology, Johns Hopkins University, Baltimore, MD USA; 10 Department of Epidemiology, Columbia University Mailman School of Public Health, New York, NY USA; 11 Department of Biostatistics and Epidemiology, The University of Oklahoma Health Sciences Center, Oklahoma, USA; 12 Department of Medicine, Stephenson Cancer Center, University of Oklahoma Health Sciences, Oklahoma City, OK USA; 13 Bioinformatics Unit, Institute for Biomedical Research INCLIVA, Valencia, Spain; 14 Universidad Autonoma de Madrid, Madrid, Spain; 15 CIBERESP (CIBER of Epidemiology and Public Health), Madrid, Spain

**Keywords:** Lymphatic cancers, Hematopoietic cancers, DNA methylation, Epigenetics, American Indians

## Abstract

**Background:**

Epigenetic alterations may contribute to early detection of cancer. We evaluated the association of blood DNA methylation with lymphatic–hematopoietic cancers and, for comparison, with solid cancers. We also evaluated the predictive ability of DNA methylation for lymphatic–hematopoietic cancers.

**Methods:**

Blood DNA methylation was measured using the Illumina Infinium methylationEPIC array in 2324 Strong Heart Study participants (41.4% men, mean age 56 years). 788,368 CpG sites were available for differential DNA methylation analysis for lymphatic–hematopoietic, solid and overall cancers using elastic-net and Cox regression models. We conducted replication in an independent population: the Framingham Heart Study. We also analyzed differential variability and conducted bioinformatic analyses to assess for potential biological mechanisms.

**Results:**

Over a follow-up of up to 28 years (mean 15), we identified 41 lymphatic–hematopoietic and 394 solid cancer cases. A total of 126 CpGs for lymphatic–hematopoietic cancers, 396 for solid cancers, and 414 for overall cancers were selected as predictors by the elastic-net model. For lymphatic–hematopoietic cancers, the predictive ability (C index) increased from 0.58 to 0.87 when adding these 126 CpGs to the risk factor model in the discovery set. The association was replicated with hazard ratios in the same direction in 28 CpGs in the Framingham Heart Study. When considering the association of variability, rather than mean differences, we found 432 differentially variable regions for lymphatic–hematopoietic cancers.

**Conclusions:**

This study suggests that differential methylation and differential variability in blood DNA methylation are associated with lymphatic–hematopoietic cancer risk. DNA methylation data may contribute to early detection of lymphatic–hematopoietic cancers.

## Introduction

Epigenetic modifications—heritable and reversible changes in the genome without changes in the DNA sequence—are involved in tumorigenesis, potentially enabling early cancer detection. Modifications in DNA methylation, the most established epigenetic measure, occur in early stages of tumor development [1] and have been associated with cancer-related biological processes including oxidative stress [2] and apoptosis [3]. Many types of human cancers show hypermethylation of regulatory regions of certain tumor-suppressor genes [4]. DNA methylation-based biomarkers have been a target for early detection of cancer [5] due to their early and frequent emergence in tumors, their high quality measurement by well-established methods, their stability over time, their presence in different body fluids, and their cell type specificity [6]. However, only two DNA methylation-based tests have received FDA approval to date, both of them for colorectal cancer screening protocols [6].

Lymphatic and hematopoietic cancers affect the blood, bone marrow, lymph, and lymphatic system tissues. They are classified as myeloid (affecting mainly blood, including leukemia) and lymphoid (affecting mainly lymph nodes) neoplasms [7]. In 2019, they were expected to account for 10% of new cancer cases diagnosed in the United States [8].

For most cancers, early detection using DNA methylation is limited by the need for biopsy and access to the target tissue. For lymphatic and hematopoietic neoplasms, blood is a much more readily available biospecimen, providing a ready opportunity to identify markers that can detect cancer in early stages of development. Global DNA hypomethylation has been associated with better clinical outcomes in acute lymphoblastic leukemia [9] and acute myeloid leukemia [10, 11], and has also been used to conduct genetic characterization for stratification of acute myeloid leukemia risk groups [12]. In addition, site-specific differential blood DNA methylation in humans has been identified in several epigenome-wide association studies for multiple myeloma [13], B-cell lymphoma [14] and chronic lymphocytic leukemia [15], and in vitro for T-acute lymphoblastic leukemia [16]. Those studies, however, compared prevalent cases to controls and lacked follow-up, which is critical both for prediction and association purposes. In addition, the number of samples or the number of CpGs included in prior studies was small.

Because blood represents the relevant target tissue for lymphatic–hematopoietic tumors, we hypothesized that DNA methylation changes in blood may have a better ability to predict these compared to solid tumors. The objective of this study was to investigate the association of blood DNA methylation with lymphatic–hematopoietic and non-lymphatic–hematopoietic (solid) tumors in the Strong Heart Study (SHS), a prospective cohort study that has followed adult men and women since 1989–1991. In addition to estimating Differentially Methylated Positions (DMPs) and Differentially Methylated Regions (DMRs), we also tested for Differentially Variable Positions (DVPs) and regions (DVRs), which are underexplored but increasingly recognized as predictors of field defects (tissue transformations that predate tumor development). We assessed replication in an independent population: the Framingham Heart Study (FHS), a prospective cohort study of adults of European ancestry in Framingham, MA followed for health outcomes for decades [17].

## Methods

### Main study population: the Strong Heart Study

**Fig. 1:**
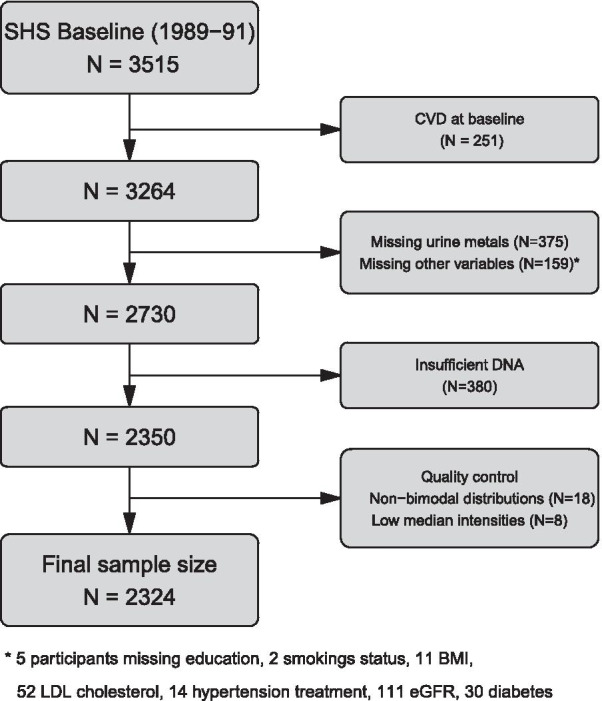
Flowchart of the included participants from the Strong Heart Study

The SHS is a prospective cohort study funded by the National Heart, Lung and Blood Institute to investigate cardiovascular diseases and risk factors in American Indian adults [18]. In 1989–1991, 4549 men and women aged 45–75 years members of 13 tribes from Arizona, Oklahoma, and North and South Dakota agreed to participate. To analyze blood DNA methylation, we had a series of exclusion criteria that were not related to the cancer outcome (Fig. 1): (1) Due to tribal request, samples from one of the tribes were not selected for DNA methylation analyses, leaving 4091 participants. (2) As we needed to use metal data to answer other research questions, participants without sufficient urine for metal determinations were excluded, leaving 3515 participants. (3) Cardiovascular disease was a primary aim for the methylation data, so participants who were free of cardiovascular disease and were not missing other variables of interest at baseline (1989–1991) were eligible for blood DNA methylation analyses (*N* = 2730). (4) Sufficient genomic DNA was available for DNA methylation analyses in 2350 participants. (5) After laboratory analyses, data from individuals without classical bimodal distribution in DNA methylation levels and from individuals with low median intensity levels were removed, leaving a total of 2324 participants for this study. These participants were similar by sociodemographic and anthropometric characteristics to the eligible participants (Table 1).

**Table 1:** Descriptive characteristics for eligible participants versus finally selected participants

	Included (*N* = 2324)	Eligible (*N* = 2730)
Age (years), median (IQR)	55.0 (49.2, 62.0)	54.9 (49.2, 62.0)
Sex (% male)	41.4	40.7
Smoking status		
% Current	38.4	37.7
% Former	32.2	33.0
BMI, median (IQR)	29.6 (26.3, 33.6)	29.7 (26.3, 33.7)
Education		
% High	58.6	59.4
% Medium	23.9	23.5
Alcohol consumption		
% Current	43.1	42.9
% Former	42.2	42.2

*IQR* interquartile range

### Participant characteristics

Trained and certified personnel collected information on sociodemographic factors, medical history, smoking status and alcohol consumption in a personal interview. Participants having smoked < 100 cigarettes in their lifetime were considered never smokers. Participants having smoked ≥ 100 cigarettes in their lifetime and smoking at the time of the interview were considered current smokers. Participants having smoked ≥ 100 cigarettes in their lifetime but currently not smoking were classified as former smokers. Current alcohol consumption was defined as any alcohol use within the past year. Former alcohol consumption was defined as no use of any alcohol during the last year but previous use of > 12 drinks of alcohol. The physical exam included anthropometric measures (height and weight), and collected fasting blood and spot urine samples.

### Cancer incidence follow-up

The SHS used tribal records, death certificates, medical records, and direct annual contact with participants and their families to assess health outcomes and vital status over time. Cancer incidence was assessed by interviews, death certificates and/or chart reviews. For these analyses, we evaluated total cancer incidence, lymphatic and hematopoietic cancer incidence (codes 200–208), and non-lymphatic and hematopoietic cancer incidence (all cancer codes minus codes 200–208, for simplicity called solid cancers). Participants with any prior history of cancer before baseline were excluded (136 for solid and 1 for lymphatic–hematopoietic cancers). We calculated follow-up from the date of baseline examination to the date of the cancer diagnosis or 31 December 2017, whichever occurred first.

### Microarray DNA methylation measurements

Details of microarray DNA methylation measurements at the baseline visit of the SHS (1989–1991) have been published elsewhere [19]. Briefly, buffy coats from fasting blood samples were collected in 1989–1991 and stored at − 70 °C. DNA from white blood cells was extracted and stored at the Penn Medical Laboratory, MedStar Health Research Institute under a strict quality-control system. In 2015, blood DNA was shipped with dry ice to the analytical laboratory at the Texas Biomedical Research Institute for DNA methylation analysis. DNA was bisulfite-converted with the EZ DNAm kit (Zymo Research) according to the manufacturer’s instructions. Bisulfite converted DNA methylation from white blood cells was measured using the Illumina MethylationEPIC BeadChip (850 K). Individuals with low detection p-values, cross-hybridizing probes, probes located in sex chromosomes and SNPs (Single Nucleotide Polymorphisms) with minor allele frequency > 0.05 were excluded. Single sample noob normalization and regression on correlated probes normalization were conducted following Illumina’s recommendations for preprocessing [20]. Blood cell proportions (CD8T, CD4T, NK cells, B cells, monocytes and neutrophils) were estimated using the R package FlowSorted.Blood.EPIC. The preprocessing resulted in data from 2324 individuals and 788,368 CpG sites in our analyses.

### Replication population: the Framingham Heart Study

The FHS is a community-based study [17]. In this study, participants from the FHS Offspring cohort, participants who attended exam cycle 8 (2005–2008, *N* = 2202) and Third Generation cohort participants who attended exam cycle 2 (2008–2011, *N* = 1455) were eligible. The study protocol was approved by the Institutional Review Board at Boston University Medical Center (Boston, MA).

Cancer was defined as the occurrence of any type of malignant tumor excluding non-melanoma skin neoplasms. Diagnoses were confirmed from pathology and laboratory reports and clinical notes. Age-specific incidence rates were compared with Connecticut Surveillance, Epidemiology, and End Results (SEER) data [21]. Participants with any prior history of cancer before the blood draw for DNA methylation measurements were excluded. Participants were followed from the time of blood collection to the time of cancer incidence (*N* = 376), which extended to December 31, 2016. These included hematological cancers (*N* = 28) and other (solid tumor) cancers (*N* = 348). Body Mass Index (BMI) was calculated as weight (kg) divided by height squared (m^2^). Current smoking (yes/no) was defined as smoking on average at least one cigarette per day during the past 12 months. Smoking pack-years was computed by multiplying the average number of cigarettes smoked per day by the number of years smoked, divided by 20. Cell type fractions of CD4T, CD8T, NK cells, monocytes and eosinophils were estimated from DNA methylation data using the Houseman method [22].

DNA samples were extracted from whole blood buffy coat samples using the Gentra Puregene DNA extraction kit (Qiagen, Venlo, Netherland) and subsequently underwent bisulfite conversion using the EZ DNA methylation kit (Zymo Research, Irvine, CA). DNA methylation levels were measured using the Illumina Infinium Human Methylation450 BeadChip (450 K). FHS Offspring cohort samples were run in two laboratory batches (batch #1 and #2). The Third Generation samples were run in batch #3. For each separate lab batch, DNA methylation beta values from Illumina GenomeStudio were further normalized using the DASEN methodology implemented in the wateRmelon R package. We used surrogate variable analyses to eliminate unwanted variation in the DNA methylation data. Beta values were regressed on batch-specific surrogate variables, and the DNA methylation residual was taken forward. The three lab batches were merged for analyses. For sample quality control, we excluded samples with a missing DNA methylation value (detection *p* > 0.01) for > 1% CpGs, poor matching of SNPs between the 65 SNPs on the Illumina 450 K array and the GWAS array, or outliers at the multi-dimensional scaling plot. For quality control at the CpG level, we excluded CpGs with methylation values missing (detection *p* value > 0.01) for > 20% of samples, as well as CpGs previously identified to map to multiple locations on the sex chromosomes, or to have an underlying SNP (minor allele frequency > 5% in European ancestry in the 1000 Genomes Project data) at the CpG site or within 10 bp of the single base extension. A total of 415,318 CpGs were retained for analyses.

### Statistical methods

#### Differentially Methylated Positions (DMPs)

Standard Cox Proportional Hazard Regression models are limited in accounting for large numbers of predictors or correlated data. Thus, we used GLMnet penalized regression, a mix between Ridge and Lasso regression in an elastic-net framework [23] which tests all CpG sites simultaneously. This approach has shown to be successful for high-dimensional methylation data [24] as well as genome-wide association studies of SNPs [25, 26]. The elastic-net penalty is controlled by the *α* parameter, where the default would be *α* = 1 (Lasso regression) and Ridge regression would be *α* = 0. Importantly, the Lasso penalty tends to select only one variable among the set of correlated variables, whereas the Ridge penalty offers more flexibility and could introduce more than one predictor from a correlated set in the models. We selected *α* = 0.05 based on the performance of the model after trying different values on the range between 0 and 1. This level of *α*, which is close to Ridge regression, has been a popular choice and has shown to work well for methylation data. The regularization path is computed for the selected penalty at a set of values as specified by the regularization parameter *λ*, which was selected using 10-folds cross-validation in our study. This model is thus also useful for avoiding genomic inflation, which is a concern in all Epigenome-Wide and Genome-Wide Association Studies. DNA methylation proportions at a given CpG (beta values) were used as predictors with age as time scale and individual entry times (age at baseline) treated as staggered entries for lymphatic–hematopoietic, solid and overall cancers. Models were adjusted for biologically relevant variables (sex, smoking status (never, former, current), BMI, blood cell counts (CD8T, CD4T, NK cells, monocytes and B cells), study region (Arizona, Oklahoma, North Dakota and South Dakota) and five genetic PCs [27]. Predictive ability was evaluated using Harrell’s concordance or C index. For replication, we ran elastic-net in the SHS restricting the CpGs to those present in 450 K (as no data from the EPIC array were available in the FHS) and we fitted an elastic-net model in the FHS population introducing the CpGs that the model selected in the SHS.

Since statistical inference based on the coefficients from the elastic-net model is unreliable given the shrinkage of the coefficients, we ran Cox proportional hazards models comparing the 90th versus the 10th percentile of DNA methylation with the CpGs selected by the elastic-net in order to report hazard ratios (HRs).

For comparison with approaches commonly used in the literature, we ran Cox proportional hazard models comparing the 90th versus the 10th percentile of DNA methylation epigenome-wide (i.e. including all CpG sites) for lymphatic–hematopoietic, solid and all cancers.

#### Protein–protein interaction network

We created lists of unique protein-coding genes from the CpGs selected by elastic-net for lymphatic–hematopoietic and solid tumors, respectively. We constructed a protein interaction network using the STRING database v11.0 [28], which provides a confidence score (from 0 to 1) to indicate the estimated likelihood that the annotated interaction between a given pair of proteins is biologically meaningful, specific and reproducible, according to the evidence derived from in-house predictions, homology transfers and externally maintained databases. We displayed a protein interaction network with Cytoscape v. 3.8.0 [29] using the yfiles Organic layout. In the resultant network, we only kept connections obtained from experimental studies, publicly available databases and text mining with a minimum confidence score of 0.3. Nodes that had no connections were excluded.

#### Differentially Methylated Regions (DMRs)

Testing differential methylation at the regional level might have several advantages as compared to the single position approach. DMRs can remove spatial redundancy by reducing the dimensionality of the often spatially correlated methylation levels and might offer increased robustness [30]. In addition, some studies have argued that DMRs might be more biologically relevant than DMPs [31, 32]. We used the R package DMRcate, which computes a kernel estimate against a null comparison to identify Differentially Methylated Regions, and ranks the DMRs by Stouffer *p* value [33]. DMRs were calculated based on the combination of the Cox regression results for individual CpGs. CpGs were annotated to the closest gene based on hg19 notation.

#### Differentially Variable Positions (DVPs) and Regions (DVRs)

We used the R package missMethyl for the DVP analysis between cases and non-cases (no survival method is available to date). The function varFit calculates a measure of variability (absolute deviation) for each CpG site and then fits a linear model to the deviations. Empirical Bayes shrinkage is applied to the residuals of the linear model to obtain robust moderated *t* statistics [34]. Multiple comparisons were accounted for using the Benjamini and Hochberg method to control for the false discovery rate (FDR) [35]. We report Log Var Ratios, which are defined as the natural log of the ratio of the absolute deviations of cancers versus non-cancers. A Log Var Ratio of log(2) would mean that the variance of one group is twice that of the second group. For the regional analysis, we used the DMRcate package.

#### Sensitivity analyses

We further adjusted the cancer models for a family history of cancer in first-degree relatives and for alcohol consumption (never, former, current) to see if the predictive ability changed. Additionally, we excluded all cases diagnosed in the first 5 years of follow-up (before 1995) to evaluate if DNA methylation could predict better cases in the near future. We analyzed lymphatic cancers (lymphomas) and hematopoietic cancers (myelomas and leukemias) separately to see if we could observe differences. Last, among the CpG sites that were selected by the elastic-net model, we repeated the Cox models adjusting for epigenetic aging instead of chronological age, using three different epigenetic aging biomarkers: the Hannum clock [36], the Horvath clock [37] and the PhenoAge [38]. The aim was to explore if some of the methylation changes might be reflecting aging.

## Results

### Descriptive analysis

Participants with incident cancer were older and more likely to be current smokers than non-cases (Table 2). Participants with incident lymphatic–hematopoietic cancers had higher BMI at baseline than solid cancers and non-cases. During follow up there were 420 new-onset cancer cases including 41 lymphatic–hematopoietic tumor cases. The mean follow-up time among participants who did not develop cancer was 26.8 years. The mean time from blood samples collection to cancer diagnosis was 14.7 years for lymphatic–hematopoietic cancers and 15.1 years for solid cancers and overall cancer. Solid cancers included 85 lung cancers, 49 breast cancers, 44 colorectal cancers, 24 kidney cancers, 23 pancreatic cancers, 22 stomach-esophagus cancers, 21 liver cancers, 15 ovarian cancers, 15 gallbladder cancers, 4 endometrial cancers, 2 thyroid cancers, and 214 other solid neoplasms (one individual might have several types of cancers).

**Table 2:** Participants’ characteristics by cancer status

	Lymphatic–hematopoietic cancer (*N* = 41)	Solid cancers (*N* = 394)	Overall cancer (*N* = 420)	No cancer (*N* = 1904)
Age (years), median (IQR)	53.2 (49.8, 59.9)	56.4 (50.5, 64.0)	56.2 (50.4, 63.7)	54.7 (49.0, 61.7)
Sex, % male	36.6	46.2	46.0	40.5
Smoking status, %				
Former	22.0	30.2	22.6	30.9
Current	46.3	47.2	46.4	36.7
BMI, median (IQR)	31.5 (26.9, 36.5)	29.0 (25.5, 33.8)	29.2 (25.7, 33.9)	29.7 (26.3, 33.5)

Medians (IQR) or percentages are shown for continuous or categorical variables, respectively

*IQR* interquartile range

### Differentially Methylated Positions

The elastic-net model for lymphatic–hematopoietic cancer selected 126 CpG sites as relevant. Among them, 10 were annotated to the gene *FAM65B*. The C index comparing the model that only included risk factors (age, sex, smoking status, BMI, blood cell counts, study region and five genetic PCs) to the model that further included DNA methylation increased from 0.5 to 0.87 (Table 3). The results from the Cox proportional hazards model for the selected CpGs by elastic-net are shown in Table S1 (Additional file 1). When considering each CpG separately, 12,342 DMPs were epigenome-wide significant at FDR < 0.05. The genomic inflation factor was 1.41 (41% of false positives, data not shown).

**Table 3:** Predictive ability of DNA methylation for lymphatic–hematopoietic, solid and overall cancers in the Strong Heart Study from the elastic-net model

	N predictors	Lymphatic–hematopoietic cancer		Solid cancers		Overall cancer	
		C index	*N* coef > 0^b^	C index	*N* coef > 0^b^	C index	*N* coef > 0^b^
Risk factors^a^	5	0.50	0	0.66	5	0.65	5
Risk factors^a^ + cell counts + genetic PCs	15	0.50	0	0.67	15	0.66	14
Risk factors^a^ + cell counts + genetic PCs + DMPs	788,383	0.87	126	0.79	396	0.79	414

*coef* coefficient, *PCs* principal components, *DMPs* Differentially Methylated Positions

^a^Age (years), smoking status (current/former/never), sex (men/women), BMI (kg/m^2^) and study center (AZ, OK, ND/SD)

^b^Variables with coef 0 are considered not to play any role in prediction

For solid cancers, the elastic-net model selected 396 CpG sites including one CpG annotated to the oncogene *LMO2* and seven CpGs annotated to smoking-related genes (*AHRR, F2RL3, PRSS23* and *GFI1*). All the CpGs annotated to smoking-related genes were inversely associated with incident lung cancer in our population (data not shown), meaning that hypomethylation in those genes would increase lung cancer risk. The C index comparing the model that only included risk factors to the model that further included DNA methylation increased from 0.66 to 0.79 (Table 3). The results from the Cox proportional hazards model for those CpGs are shown in Table S2 (Additional file 1). No DMPs were found by the traditional epigenome-wide association study (EWAS) approach at 0.05 FDR significance level.

For overall cancer, the elastic-net model selected 414 CpG sites of which 250 were also selected for solid tumors and two for lymphatic–hematopoietic cancers. The C index increased from 0.66 to 0.79 after including DNA methylation in the model (Table 3). The results from the Cox proportional hazards model for those CpGs are shown in Table S3 (Additional file 1). No DMPs were found by the traditional epigenome-wide association study (EWAS) approach at 0.05 FDR significance level.

### Replication

Replication results of DNA methylation and cancer in the FHS are shown in Table 4. For lymphatic–hematopoietic cancers, the C index for a model including only risk factors in the FHS (age, sex, BMI and smoking status) was 0.76, and it increased to 0.89 when further including CpG sites selected by the SHS model as well as cell counts (Table 4). For solid tumors, the C index for a model including only risk factors in the FHS was 0.69, and it increased to 0.75 when further including the CpGs selected by the SHS model and cell counts (Table 4). For overall cancers, the C index when only including risk factors in the FHS was 0.69, and it increased to 0.74 when further including the CpGs selected by the SHS model and cell counts (Table 4). The results from the Cox proportional hazards model for those CpGs for lymphatic–hematopoietic, solid and overall cancers are show in Additional file 1 (Tables S1, S2 and S3, respectively). 28 CpGs for lymphatic–hematopoietic, 54 for solid and 37 for overall cancers had HRs in the same direction as in the SHS.

**Table 4:** Replication: predictive ability in the Framingham Heart Study (450 K) of the CpGs selected in the Strong Heart Study for lymphatic–hematopoietic, solid and overall cancers

	N predictors	Lymphatic–hematopoietic cancer		Solid cancers		Overall cancer	
		C index	*N* coef > 0^b^	C index	*N* coef > 0^b^	C index	*N* coef > 0^b^
Risk factors^a^	5	0.76	5	0.69	4	0.69	5
Risk factors^a^ + cell counts	11	0.79	10	0.69	11	0.69	10
Risk factors^a^ + cell counts + DMPs	132/379/399^c^	0.89	62	0.75	34	0.74	32

*coef* coefficient, *PCs* principal components, *DMPs* Differentially Methylated Positions

^a^Age (years), smoking status (current/former/never), sex (men/women) and BMI (kg/m^2^)

^b^Variables with coef 0 are considered not to play any role in prediction

^c^For lymphatic–hematopoietic and overall cancers, among the 125 CpGs selected by elastic-net in the Strong Heart Study (restricting to CpGs included in 450 K), 123 were present in the Framingham Heart Study. In addition, the four risk factor variables and the five cell count variables were included in the elastic-net model: a total of 132 variables. For solid cancers, 373 CpGs were selected in the Strong Heart Study, 370 being present in the Framingham Heart Study. With the four risk factor variables and the five cell count variables, a total of 379 variables were included. For overall cancers, 395 CpGs were selected in the Strong Heart Study, 390 being present in the Framingham Heart Study. With the four risk factor variables and the five cell count variables, a total of 399 variables were included

### Protein–protein interaction network

**Fig. 2:**
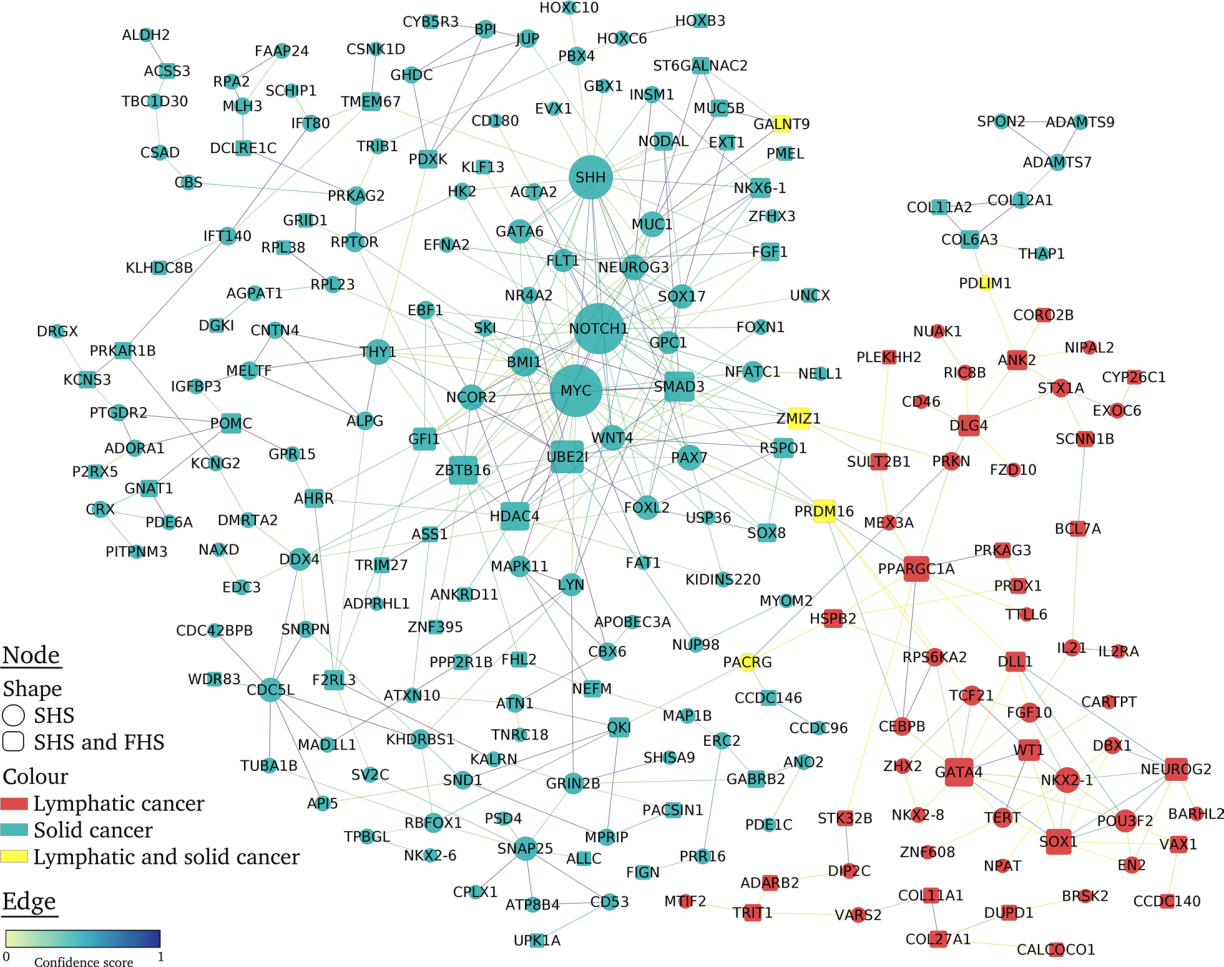
Protein–Protein interaction network of Differentially Methylated Positions in lymphatic–hematopoietic cancer. The circle nodes indicate Differentially Methylated Positions in the Strong Heart Study and the square nodes those replicated in the Framingham Heart Study. The red nodes indicate Differentially Methylated Positions for lymphatic–hematopoietic cancers, the blue nodes for solid cancers and the yellow nodes for all cancers. The size of the nodes is proportional to the number of connections. The edges indicate confidence scores for interactions from 0 to 1

When restricting the SHS analyses to 450 K, 126 and 373 CpGs were selected for lymphatic–hematopoietic and solid tumors, respectively, which included 442 unique genes. Among those, 218 were ncRNA genes or non-connected nodes. Thus, a network with 224 nodes and 398 interactions was obtained (Fig. 2). From 57 lymphatic–hematopoietic nodes identified in the SHS, 26 were also identified in the FHS population, being *GATA4, SOX1* and *PPARGC1A* the most connected (11, 9 and 9 interaction, respectively). For 162 solid cancer nodes identified in the SHS, 50 nodes were also identified in the FHS population, being *MYC*, *NOTCH1* and *SHH* the most connected nodes in the network (> 20 connections). The remaining 5 nodes (*PRDM16*, *GALNT9*, *PACRG*, *PDLIM1* and *ZMIZ1)* were reported in both lymphatic–hematopoietic and solid tumors. Details of the network are included in Additional file 2.

### Differentially Methylated Regions (DMRs)

**Fig. 3:**
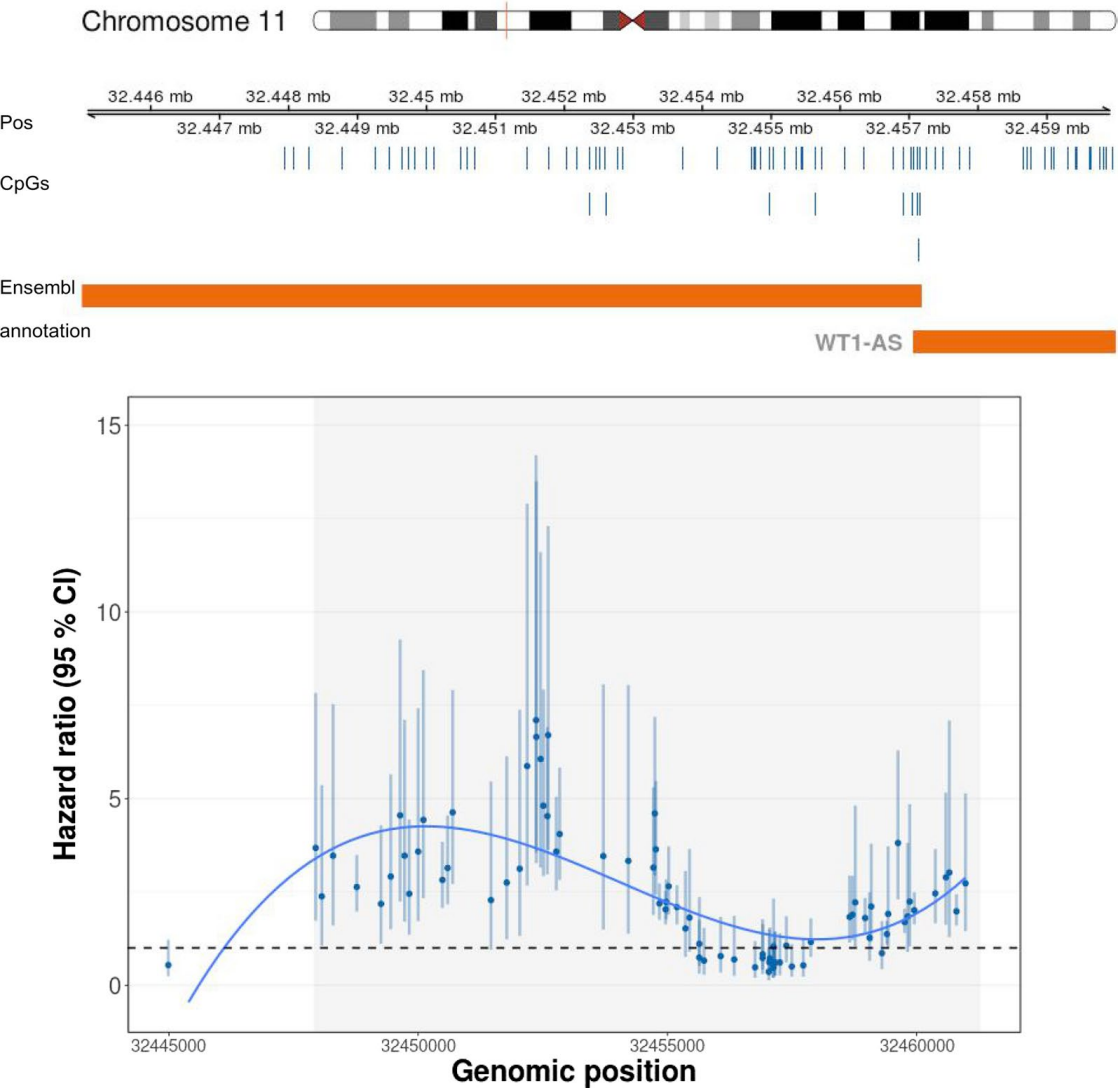
Differentially methylated region for lymphatic–hematopoietic cancer. Hazard ratios (95% confidence intervals) and genomic location of the top 2 differentially methylated region for lymphatic–hematopoietic cancers including 41 CpG sites. Orange bars represent overlapping promoters. Locations of CpGs of the differentially methylated region in the chromosome are represented by blue vertical bars above the overlapping promoters. The grey area in the plot represents the differentially methylated region

We found 159 DMRs for lymphatic–hematopoietic cancers. The top 15 are shown in Table 5. No DMRs were found for overall or solid tumors. The number of CpGs included in the DMRs for lymphatic–hematopoietic cancers ranged from 4 to 41. The region 24910562: 24912385 (chromosome 6), annotated to the gene *FAM65B*, was the top DMR, including 20 CpGs. The top two DMR, reflecting 41 CpG sites, was annotated to the gene *WT1*. Figure 3 shows the tendency of the associations of the individual CpGs within this DMR; a bump of highly hypermethylated CpG sites followed by a flat area with no significant sites and another hypermethylation bump is observed.

**Table 5:** Top 15 Differentially Methylated Regions for lymphatic–hematopoietic cancers

Positions	Chr	Width	N CpGs	Stouffer *p* value	Gene	Function	Overlapping promoters
24910562: 24912385	chr6	1824	20	2.30E−28	*FAM65B*	Inhibits the proliferation of human leukemic T cells	FAM65B-001
32447944: 32455735	chr11	7792	41	2.28E−23	*WT1*	Oncogene in Acute Myeloid Leukemia	WT1-001, WT1-005, WT1-002, WT1-AS-001, WT1-AS-201, WT1-AS-005, WT1-009, WT1-004, WT1-AS-003, WT1-AS-004, WT1-AS-002, WT1-AS-006, WT1-006, WT1-003
31159558: 31160393	chr16	836	6	5.77E−16	*PRSS36*	Serine protease (cleaves peptide bonds in proteins)	PRSS36
73565440: 73565966	chr10	527	5	4.22E−09	*CDH23*	Encodes calcium dependent cell–cell adhesion glycoproteins. Might be associated with breast cancer	CDH23, SNORA71, SNORA17
159869223: 159870915	chr1	1693	10	1.28E−08	*CFAP45*	Uncharacterized function	snoU13, Y_RNA, SCARNA16, SNORD112, SNORA63, U3, SNORA51, SNORA25, SNORD59, SCARNA20, SNORA67, U6, SNORA70, SNORA77, SNORA26, SNORA72, U8, SNORA31, SNORA40, CCDC19, hsa-mir-4259, ACA64, SNORD78, snoU109, SNORD60, SNORD116
52995053: 52995634	chr12	582	7	1.56E−08	*KRT72*	Structural integrity of epithelial cells	RP11-641A6.2, KRT72, snoMe28S-Am2634
30476089: 30477270	chr22	1182	15	3.62E−08	*HORMAD2*	Meiotic prophase quality control. Associated with lung cancer	HORMAD2, CTA-85E5.10
149112318: 149113196	chr7	879	6	5.09E−08	*ZNF777*	Zinc finger protein. Nucleic acid binding	None
42951711: 42952369	chr5	659	4	1.21E−07	*LOC648987*	Uncharacterized function	SNORA27, SNORA68, RPS23P5, SNORA57, SNORA76, 7SK, SNORD45
157164556: 157165335	chr1	780	5	2.26E−07	*ETV3*	Transcriptional repressor associated to dendritic cell tumor	snoU13, Y_RNA, SCARNA16, SNORD112, SNORA63, U3, SNORA51, SNORA25, SNORD59, SCARNA20, SNORA67, U6, SNORA70, SNORA77, SNORA26, SNORA72, U8, SNORA31, SNORA40, SNORD64, ACA64, SNORD78, snoU109, SNORD60, SNORD116
49726500: 49727110	chr12	611	5	2.28E−07	*C1QL4*	Tumor necrosis factor	C1QL4, snoMe28S-Am2634
375248: 375830	chr10	583	5	2.83E-07	*DIP2C*	Mutations in breast and lung cancer (potential diagnosis target)	DIP2C
157182707: 157187341	chr2	4635	22	3.92E−07	*NR4A2*	Transcription factor. Potential therapeutic target for gastrointestinal cancer	5S_rRNA, SNORA4, SNORD11, SNORD51, SNORA41, SCARNA6, SNORD39, SNORA75, ACA59, SNORA48, NR4A2, SNORA43, SNORA1, Vault
76803270: 76803925	chr10	656	5	6.10E−07	*DUPD1*	Dual specificity phosphatase	SNORA71, SNORA17, DUPD1
64253534: 64253818	chr3	285	6	1.19E−06	*PRICKLE2*	Related to WNT signaling pathways	U7, SNORD77, SNORA33, SNORA81, SNORD66, SNORD2, SNORD5, SNORD38, SNORD63, PRICKLE2, Metazoa_SRP, SNORA18

Model adjusted for age, smoking status (never, former, current), sex (male/female), BMI (kg/m^2^) Houseman cell proportions (CD8T, CD4T, NK, B cells and monocytes), five genetic PCs and study center (Arizona, Oklahoma or Dakota)

### Differentially Variable Positions (DVPs) and Regions (DVRs)

**Fig. 4:**
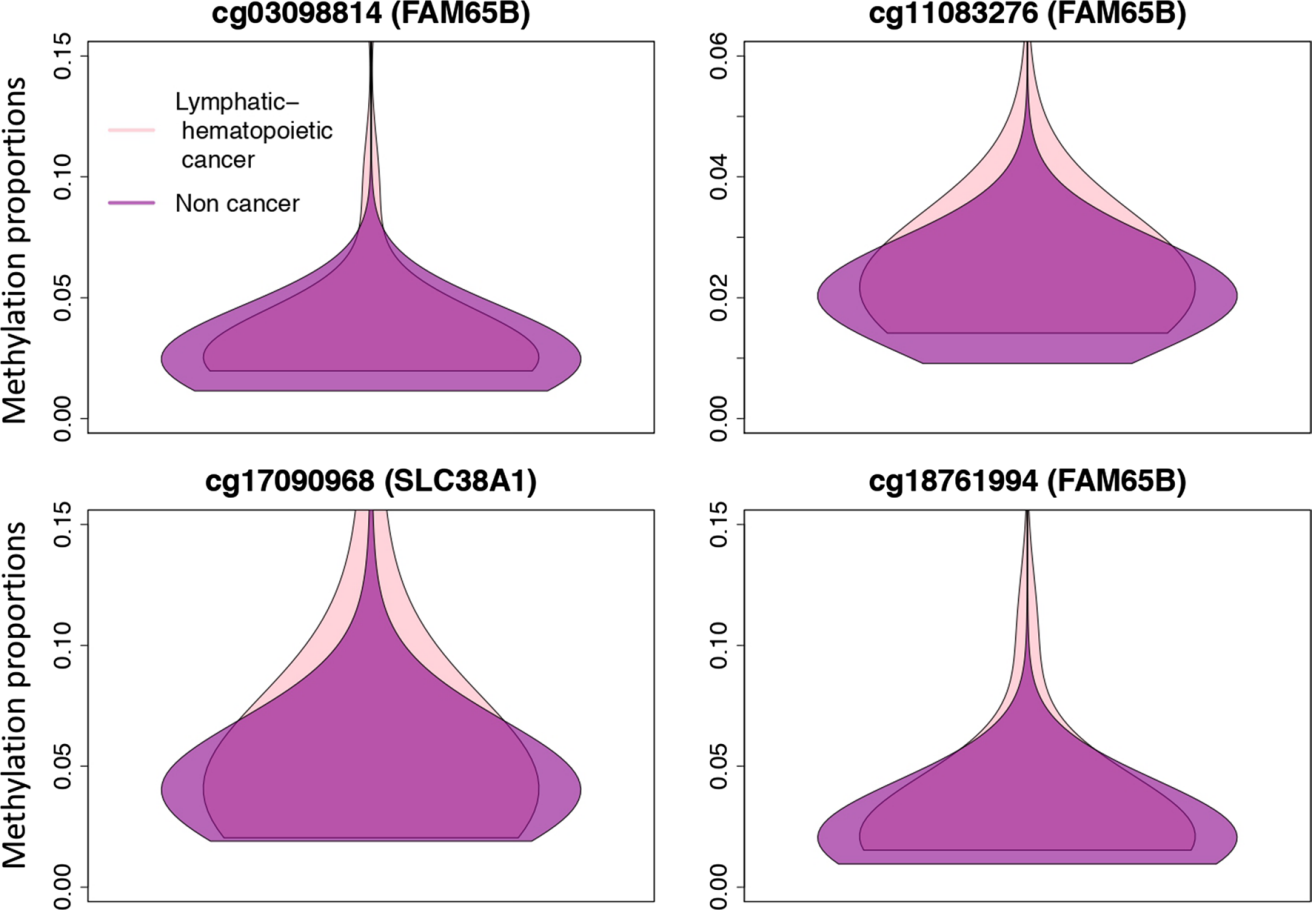
Violin plots for lymphatic–hematopoietic cancer. Distribution of the methylation proportions of lymphatic–hematopoietic cancers versus non-cases for the top four Differentially Variable Positions

At a 0.05 FDR significance level, we found 12,967 DVPs for lymphatic–hematopoietic (Table 6 shows top 15), 7 for solid (Table 7), and 9 for all cancers (data not shown). There were five common DVPs for overall and solid tumors annotated to *CCDC92, AQP12B, GFI1*, *XIRP2* and *SPRY2* genes. Other DVPs associated to solid neoplasms (Table 7) were annotated to *TBC1D12* and *MTOR* genes. The violin plots in Fig. 4 show the distribution of the methylation proportions for lymphatic–hematopoietic cancer cases versus non-cases for the top 4 DVPs. The Log Var Ratios of the top 15 DVPs for lymphatic–hematopoietic cancers range between 1.57 and 2.22, indicating the group variance is between 5 and 9 times higher (log(5) = 1.6, log(9) = 2.2) in lymphatic–hematopoietic cancer cases compared to non-cases (Table 6). 106 of the 152 CpGs selected by elastic-net were DVPs as well. We found 432 DVRs for lymphatic–hematopoietic cancers (Table 8 shows top 15); 78 were DMRs as well.

**Table 6:** Top 15 Differentially Variable Positions for lymphatic–hematopoietic cancers

CpG	Chr	Gene	Function	In 450 k	Log Var Ratio	*p* value	FDR
cg03098814	chr6	*FAM65B*	Inhibits the proliferation of human leukemic T cells	Yes	2.22	1.31E−22	1.03E−16
cg11083276	chr6	*FAM65B*	Inhibits the proliferation of human leukemic T cells	Yes	1.97	3.61E−21	1.42E−15
cg17090968	chr12	*SLC38A1*	Sodium-dependent amino acid transporter. Mediates the saturable, pH-sensitive and electrogenic cotransport of glutamine and sodium ions	Yes	1.67	1.08E−19	2.85E−14
cg18761994	chr6	*FAM65B*	Inhibits the proliferation of human leukemic T cells	Yes	2.15	1.78E−19	3.52E−14
cg17757602	chr5	*Intergenic*	Uncharacterized	Yes	2.02	4.34E−19	6.84E−14
cg11211942	chr15	*Intergenic*	Uncharacterized	No	1.92	1.26E−17	1.66E−12
cg19936032	chr6	*FAM65B*	Inhibits the proliferation of human leukemic T cells	Yes	2.04	2.34E−17	2.64E−12
cg02915015	chr6	*FAM65B*	Inhibits the proliferation of human leukemic T cells	Yes	2.04	1.15E−16	1.13E−11
cg14536812	chr12	*Intergenic*	Uncharacterized		1.84	3.60E−16	3.15E−11
cg08576643	chr6	*FAM65B*	Inhibits the proliferation of human leukemic T cells	Yes	2.15	4.26E−16	3.36E−11
cg17896599	chr6	*FAM65B*	Inhibits the proliferation of human leukemic T cells	No	2.15	6.59E−16	4.73E−11
cg01726103	chr6	*FAM65B*	Inhibits the proliferation of human leukemic T cells	Yes	1.91	8.37E−16	5.50E−11
cg24698979	chr17	*ARHGAP23*	Increases p53 proto-oncogene’s transactivity	No	1.57	1.09E−15	6.58E−11
cg18368658	chr15	*CHST14*	Regulates proliferation and neurogenesis of neural progenitor cells	No	1.60	1.62E−15	9.12E−11
cg14216285	chr6	*LINC01623*	Uncharacterized	Yes	1.79	2.20E−15	1.16E−10

Log Var Ratios: Natural log of the ratio of the absolute deviations of cancers versus non-cancers. A Log Var Ratio of log(2) would mean that the variance of one group is twice that of the second group

Model adjusted for age, smoking status (never, former, current), sex (male/female), BMI (kg/m^2^), Houseman cell proportions (CD8T, CD4T, NK, B cells and monocytes), five genetic PCs and study center (Arizona, Oklahoma or Dakota)

**Table 7:** Differentially Variable Positions for solid cancers

CpG	Chr	Gene	Function	In 450 k	Log Var Ratio	*p* value	FDR
cg21902846	12	*CCDC92*	DNA repair and reduction/oxidation reactions	No	0.91	1.22E−11	9.65E−06
cg23070169	2	*XIRP2*	Associated to Alzheimer’s disease and Down syndrome	No	− 0.64	5.90E−09	0.0022
cg26292116	2	*AQP12B*	Migration, invasion and proliferation of human breast tumor cells	No	− 0.33	8.44E−09	0.0022
cg13911116	1	*MTOR*	Its activation promotes tumor growth and metastasis, many MTOR inhibitors have been approved to treat human cancers	Yes	0.28	1.05E−07	0.021
cg21383151	10	*TBC1D12*	Mutations suggested to be related to bladder cancer	Yes	0.41	1.55E−07	0.023
cg08598861	13	*SPRY2*	Regulates metastatic potential and differentiation in several cancers	No	− 0.59	1.74E−07	0.023
cg18146737	1	*GFI1*	Significant role in development of lung cancer and prostate cancer and tumor suppressor gene in colorectal cancer	Yes	0.53	3.37E−07	0.038

Log Var Ratios: Natural log of the ratio of the absolute deviations of cancers versus non-cancers. A Log Var Ratio of log(2) would mean that the variance of one group is twice that of the second group

Models adjusted for age, smoking status (never, former, current), sex (male/female), BMI (kg/m^2^), cell proportions (CD8T, CD4T, NK, B cells and monocytes), five genetic PCs and study center (Arizona, Oklahoma or Dakota)

**Table 8:** Top 15 Differentially Variable Regions for lymphatic–hematopoietic cancers

Positions	Chr	Width	N CpGs	Stouffer *p* value	Gene	Function	Overlapping promoters
24910562: 24912896	chr6	2335	21	1.55E−51	*FAM65B*	Inhibits the proliferation of human leukemic T cells	FAM65B-001
32447944: 32456069	chr11	8126	42	7.89E−33	*WT1*	Oncogene in Acute Myeloid Leukemia	WT1-001, WT1-005, WT1-002, WT1-AS-001, WT1-AS-201, WT1-AS-005, WT1-009, WT1-004, WT1-AS-003, WT1-AS-004, WT1-AS-002, WT1-AS-006, WT1-006, WT1-003
748992: 749620	chr20	629	9	1.66E−23	*SLC52A3*	Predictive and prognostic biomarker in esophageal cancer	SLC52A3-001, SLC52A3-004, SLC52A3-003
36147197: 36150135	chr20	2939	48	1.08E−17	*BLCAP*	Regulates cell proliferation and coordinate apoptosis and cell cycle progression. Associated to bladder and cervical cancers	NNAT-001, NNAT-002, BLCAP-009, BLCAP-010, BLCAP-005
27141388: 27144595	chr7	3208	28	2.55E−17	*HOXA2*	Aberrant expression associated to several cancers	HOXA2-001
16829666: 16830859	chr19	1194	10	8.58E−12	*NWD1*	Suggested causal role in dysregulation of androgen receptor signaling during prostate cancer progression	NWD1-002, NWD1-001, NWD1-006
42950995: 42952369	chr5	1375	5	6.85E−11	*LOC648987*	Uncharacterized function	-
157164556:157165335	chr1	780	5	1.72E−10	*ETV3*	Transcriptional repressor associated to dendritic cell tumor	-
13120555: 13123217	chr19	2663	7	3.50E−10	*NFIX*	DNA-binding transcription factor activity	NFIX-009, NFIX-008
121624862: 121625735	chr2	874	6	2.07E−09	*GLI2*	Zinc finger protein thought to play a role in embryogenesis	RP11-297J22.1-001
36665826: 36667782	chr17	1957	10	2.23E−09	*ARHGAP23*	Increases p53 proto-oncogene’s transactivity	-
46997630: 46999840	chr19	2211	19	1.03E−08	*PPP5D1*	MAPK signaling pathway. Abnormal MAPK signaling may lead to uncontrolled cell proliferation and resistance to apoptosis	AC011484.1-201, PNMAL2-201, PNMAL2-004, PNMAL2-001
42431109: 42433041	chr17	1933	8	1.68E−08	*FAM171A2*	Associated to ceroid lipofuscinosis	GRN-022
78526804: 78527410	chr15	607	5	3.19E−08	*ACSBG1*	Associated to malignant ovarian surface epithelial-stromal neoplasm and ovary epithelial cancer	ACSBG1-001, ACSBG1-201, ACSBG1-012, ACSBG1-014, ACSBG1-002, ACSBG1-008, ACSBG1-003
17603531: 17604184	chr17	654	6	3.55E−08	*RAI1*	Transcriptional regulator of the circadian clock components. Chromatin remodeling	-

Models adjusted for age, smoking status (never, former, current), sex (male/female), BMI (kg/m^2^), Houseman cell proportions (CD8T, CD4T, NK, B cells and monocytes), five genetic PCs and study center (Arizona, Oklahoma or Dakota)

### Sensitivity analyses

Adjustment for cancer family history or alcohol consumption made no changes in the C index of the predictive models. After excluding five cases of lymphatic–hematopoietic cancers diagnosed before 1995, the C index dropped from 0.85 to 0.75. The C index did not change when excluding 33 cases of solid cancers that were diagnosed before 1995. A model including 19 cases of lymphatic cancers had a C index of 0.83, with seven CpGs being selected. A model including 20 cases of hematopoietic cancers had a C index of 0.94, with 184 CpGs being selected (including the gene *FAM65B* selected several times). Adjustment for any of the three epigenetic aging biomarkers did not change the results as compared to the adjustment for chronological aging (data not shown).

## Discussion

Differential methylation at a number of CpGs and regions was associated with the incidence of lymphatic–hematopoietic, solid, and overall cancers. The strongest epigenetic signals were apparent for lymphatic–hematopoietic cancers, and the increase in prediction ability was substantially higher for lymphatic–hematopoietic cancers compared to the other cancers. Of note, improvement in event prediction for lymphatic–hematopoietic cancer cases was due to cases occurring during early follow up and may reflect blood DNA methylation predicting subclinical disease. The improvement in predictive ability for lymphatic–hematopoietic cancers as well as the direction of association for several CpGs was replicated in the FHS, an independent population of white men and women from Framingham, MA. Whereas several signals showed to be robust across both populations, other CpGs were not replicated in the FHS and some of them had opposite directions of association. Given that DNA methylation is highly influenced by environmental and genetic factors, population-specific effects for methylation sites might exist [39]. Our results support stronger and more robust signals for hematopoietic than for lymphatic cancers. This might be related to the specificity of the blood tissue.

The issue of genomic inflation and the spatial redundancy among correlated CpGs may make DMRs a more appropriate and robust approach than DMPs calculated by individual models for each CpG [30]. DMR approaches, however, remain spatially defined and do not include non-contiguous CpG sets [40]. For this reason, studying all CpG sites together in the same model might be more appropriate than studying them separately. When introducing all the CpG sites in the elastic-net model for lymphatic–hematopoietic cancers, only 126 were selected, in contrast to the 12,342 sites identified in the traditional EWAS DMP modeling. One possible reason for this large drop in the number of CpGs is the reduction in redundancy among correlated methylation across multiple CpGs, either due to spatial correlation or to methylation-level interactions on disease risk.

Our results are consistent with those from a case–control study in a population from three different cities in the US [15] that studied genome-wide DNA methylation changes in chronic lymphocytic leukemia. They found cancer-related hypermethylation in HOX gene clusters. Two of our DVRs and a DMR for lymphatic–hematopoietic cancers were annotated to genes *HOXA2* and *HOXA-AS3* and overlapped with promoters of the HOX family, whose aberrant expression levels have been related to several cancers [41–45]. The second top both DMR and DVR in our study (including 41 CpG sites) was annotated to *WT1,* an oncogene in acute myeloid leukemia. Another top DMR was annotated to *PRICKLE2. WT1* and *PRICKLE2* genes are part of the WNT signaling pathway. Hypermethylation in genes related to WNT signaling pathway was also found in the aforementioned case–control study [15]. Moreover, mutations in *WT1* have been recurrently identified in acute myeloid leukemia and associated with poor prognosis and chemotherapy resistance [46, 47]. The DMRs annotated to *HOXA2* and *WT1* in our study were hypermethylated, consistently with the case–control study [15].

Despite limitations in methods for prospective analyses, DVPs have previously been shown to be valuable for early cancer detection [30]. Differential variability detected field defects (tissue transformations that may predate cancer) in breast [48] and cervical [49] cancers. In our study, differential variability was associated with lymphatic–hematopoietic cancer with an extremely large number of DVPs identified. In addition, 96 of the 126 CpGs selected by the elastic-net models for lymphatic–hematopoietic cancers were also DVPs, reflecting the importance of variability in methylation for the occurrence of these tumors. An example of the aforementioned spatial redundancy can be seen in our DVP results (Table 6), where most of the top CpGs are annotated to *FAM65B*. These DVPs are encompassed into a single DVR in chromosome 6 annotated to *FAM65B* in Table 8. The gene *FAM65B* is repeatedly showing as differentially methylated and differentially variable in our study; furthermore, seven of the selected CpGs by the elastic-net model were annotated to this gene, suggesting its importance for lymphatic–hematopoietic cancers. *FAM65B*’s function is to control the proliferation of transformed and primary T cells [50]. In transformed T lymphocytes, forced expression of *FAM65B* blocks their mitosis, leading to G2 cell cycle arrest and apoptosis. In a public database including 75,000 individuals with methylation and cancer data [51], the CpG sites from chromosome 6 annotated to gene *FAM65B* had more variability in acute myeloid leukemia cases than in controls, which is consistent with our results. Research is needed to understand the potential role of this gene in lymphatic–hematopoietic cancers. Other genes to which DVRs were annotated were also related to the lymphatic or hematopoietic systems such as the gene *ETV3,* associated to dendritic cell tumor, which develops from cells of the immune system, typically beginning in the lymph system [52].

Differential variability might also be relevant for solid cancers. We found a DVP annotated to *MTOR*, which regulates cell growth, survival, metabolism and immunity. Activation of *MTOR* promotes tumor growth and metastasis, and many *MTOR* inhibitors have been developed to treat cancer [53]. Some of them have already been approved and are being used with modest success, while others are still being evaluated in clinical trials [54]. Other DVPs for solid cancers were annotated to genes related to bladder (*TBC1D12*), breast (*AQP12B*) or lung, prostate and colorectal (*GFI1*) cancers. *GFI1* has been identified as a potential therapeutic target for interfering with inflammation-induced colorectal cancer progression and spread [55]. Of note, several CpGs annotated to smoking-associated genes were identified as predictive of solid cancers in both the SHS and the FHS (*AHRR* and *F2RL3*) or only in the SHS (*PRSS23* and *GFI1).* These genes were individually associated with lung cancer in the SHS and might be predictive of other specific solid smoking-related cancers as well.

In addition, the protein interaction network showed highly connected nodes in both populations that have previously been related to cancer. For instance, the hub nodes *MYC*, *NOTCH1* and *SHH* have been associated to different types of cancer [56]. The *GATA4* gene encodes a member of a zinc-finger transcription factors family and alterations in gene expression in this gene have been associated with cancer [57]. Methylation in *PPARGC1A* gene was reported to predict cancer incidence [58]. The common nodes for solid and lymphatic–hematopoietic cancers have also been previously associated to cancer, for instance *PRDM16* was related to acute myeloblastic leukemia [59]. Those highly connected nodes could be key factors for lymphatic–hematopoietic cancers development. Additional experimental research is needed to confirm the biological relevance of the findings.

This study has several limitations. First, we only have 41 cases of lymphatic–hematopoietic cancers, and we might lack power to detect signals for lymphatic and hematopoietic cancers separately. Second, we might not have been able to capture all risk factors associated with some of these tumors (e.g., data on Epstein–Barr virus infection, a risk factor for Hodgkin lymphoma). Also, the C index measure has shown to be problematic in some settings. Training a new model different to that of the discovery set might overestimate C index in replication sets [60]. At the same time, using the model trained on the discovery set on the replication set might lead to underestimation of the C index due to differences in biological factors between cohorts [60]. The development of more appropriate predictive accuracy methods for replication sets needs further investigation. Non-fatal cancer data in the SHS might be incomplete, as no linkage between the SHS cancer data and cancer registry data has been conducted to date. However, the lymphatic–hematopoietic cancer diagnosis is very specific and it is unlikely that the reported cases are incorrectly classified. On the other hand, this study has several strengths which include having comprehensive methylation in one of the largest microarrays available nowadays (Infinium methylationEPIC), the high quality of the study protocols, the availability of data to account for potential confounders, the innovative statistical methods and the replication in an independent population with a large sample size. Moreover, this is the first prospective study evaluating DNA methylation in lymphatic–hematopoietic cancers (including almost 30 years of follow-up).

## Conclusions

In conclusion, this study supports that differential methylation and differential variability in methylation are associated with lymphatic–hematopoietic cancers. Blood DNA methylation data could improve early detection of cancer beyond known risk factors. The identified DNA methylation markers may not only constitute a precision medicine tool for the early identification of blood cancers in adults, but may also help elucidate mechanisms that can inform prevention and treatment.

## Supplementary Information

**Additional file 1**. Hazard ratios (95% CIs) for lymphatic-hematopoietic, solid and overall cancers in the Strong Heart Study and the Framingham Heart Study.

**Additional file 2**. Network nodes and network edges for the protein-protein interaction network.

## Data Availability

The data underlying this article cannot be shared publicly in an unrestricted manner due to limitations in the consent forms and in the agreements between the Strong Heart Study tribal communities and the Strong Heart Study investigators. The data can be shared to external investigators following the procedures established by the Strong Heart Study, available at https://strongheartstudy.org/. All analyses were conducted in R version 3.6.2 and all packages used are freely available in the CRAN repository.

## Abbreviations

CpG: Cytosine–guanine dinucleotide
SHS: Strong Heart Study
FHS: Framingham Heart Study
DMP: Differentially methylated position
DMR: Differentially methylated region
DVP: Differentially variable position
DVR: Differentially variable region
SNPs: Single nucleotide polymorphism
HRs: Hazard ratio
